# Knowledge, attitude and practice toward diabetes among the public in the Kingdom of Saudi Arabia: a cross-sectional study

**DOI:** 10.3389/fpubh.2024.1326675

**Published:** 2024-04-17

**Authors:** Bashayer Ebraheem Al-Wagdi, Mohammed Khaled Al-Hanawi

**Affiliations:** ^1^Department of Health Services and Hospital Administration, Faculty of Economics and Administration, King Abdulaziz University, Jeddah, Saudi Arabia; ^2^Physical Therapy Department, Ahad Rafidah General Hospital, Abha, Saudi Arabia; ^3^Health Economics Research Group, King Abdulaziz University, Jeddah, Saudi Arabia

**Keywords:** knowledge, attitude, practice, diabetes, Saudi Arabia, socio-demographic

## Abstract

**Background:**

The increasing adoption of sedentary lifestyles and cultural shifts has fostered unhealthy habits and decreased physical activity, consequently exacerbating the prevalence of diabetes. Diabetes is currently one of the top 10 diseases worldwide, contributing significantly to both mortality and morbidity. Since diabetes hinges on self-care, possessing the right knowledge, attitude, and habits related to the disease is paramount. This study, therefore, aims to examine the knowledge, attitude, and practice of diabetes among the population of Saudi Arabia.

**Methods:**

The study utilized data from a cross-sectional study conducted via an online self-reported questionnaire among the general population of Saudi Arabia. The study primarily used univariate and multivariable regression data analyses. Univariate analysis was employed to compile social and demographic statistics frequencies, while One-way analysis of variance (ANOVA) was used to assess mean differences in knowledge, attitudes, and practices scores. Furthermore, a multivariable linear regression analysis was executed to identify factors associated with knowledge, attitudes, and practices.

**Results:**

The mean score for diabetes knowledge was 17.79 (SD = 5.39, range: 0–29), with an overall accuracy rate of 61.34%. The mean attitude score for diabetes was 2.33 (SD = 1.91, range: 0–7), while the mean score for diabetes practices was 2.58 (SD = 1.28, range: 0–4). The multivariate analyses reveal distinct variations in knowledge, attitudes, and practices of diabetes among participants based on their gender, education, marital status, income, diabetes patient status, and having a medical field-related education.

**Conclusion:**

High knowledge scores do not necessarily equate to positive attitudes and practices related to diabetes. There is need for intensified care and the implementation of specialized educational programs that emphasize the importance of having the right attitude and engaging in the good diabetes practices.

## Introduction

Over the years, the prominence of non-communicable diseases (NCDs) has remained a global concern. These diseases represent chronic illnesses that are characterized by their non-infectious and non-contagious nature, encompassing health conditions such as cardiovascular diseases, various forms of cancer, diabetes, and chronic respiratory diseases (1, 2). Demographic, economic, and environmental shifts, along with the COVID-19 pandemic, have exacerbated the prevalence of NCDs. COVID-19 has notably increased the susceptibility of individuals with NCDs to severe illness and mortality (3). In 2019, NCDs accounted for approximately 74% of the total global mortality, amounting to an estimated 40 million deaths (3). This highlights the global need for strategies in healthcare management aimed at reducing the prevalence of NCDs.

Diabetes is among the NCDs increasingly presenting challenges to countries, regardless of their developmental stage (4). Constituting a chronic metabolic disorder characterized by insufficient insulin production or the body’s ineffective use of insulin, diabetes ranks among the top 10 diseases contributing to mortality and morbidity (5, 6). An estimated 5 million deaths in the global population aged 20–79 were linked to diabetes in 2015 (7). The high mortality rates can be ascribed to the numerous complications associated with diabetes, including brain diseases, renal failure, vision impairment, cardiovascular problems, and limb amputations (8). This is exacerbated by the growing trend toward sedentary lifestyles and cultural shifts, which have introduced unhealthy habits and reduced physical activity (9, 10). Prioritizing the mitigation of risk factors is crucial in addressing the prevalence of diabetes.

Existing literature shows high diabetes prevalence among older individuals and those with lower education, whereas it is more common among individuals with higher incomes. The aging process reduces physical activity and weakens the immune system, thereby presenting healthcare access challenges and increasing the disease burden among the older adult (11, 12). Hence, diabetes prevalence differs among age groups, with the older adult experiencing a greater risk in contrast to younger individuals. In terms of education, individuals with lower levels of education may encounter increased exposure to risk factors and greater susceptibility to diabetes in comparison to those with higher levels of education (13–15). Education plays a vital role in raising awareness, but individuals with lower levels of education tend to have limited access to information (16, 17). Nonetheless, owing to sedentary lifestyles, numerous studies suggest a higher prevalence of diabetes among individuals with higher income levels (18, 19).

Despite specific socio-economic traits exhibiting higher prevalence, diabetes remains a burgeoning global public health challenge, profoundly affecting the population’s quality of life and well-being. The diabetic patient population is projected to exceed 700 million by 2045 (20). These statistics underscore the mounting pressure that the healthcare system will confront, leading to escalated healthcare costs, including both out-of-pocket expenses and insurance expenditures (21–23). Urgent action is required to implement strategies that can effectively address the prevalence, treatment, and management of diabetes. Since misconceptions about NCDs such as diabetes can increase the risk of complications, a sound understanding of the disease is essential for effective management. Insufficient knowledge, attitude, and practice regarding diabetes frequently correlate with unfavorable health outcomes (24). Patients well-informed about diabetes and its complications are more likely to seek appropriate treatment and healthcare (25).

A diagnostic tool employed to assess participants’ understanding, attitudes, and behaviors related to a specific phenomenon is referred to as a knowledge, attitude, and practice (KAP) study (26). It explores behaviors and practices by gathering data on what is understood, believed, and enacted in connection to a specific subject (27). KAP studies on diabetes delineate patient characteristics and the factors influencing their diabetes-related knowledge, attitudes, and practices (28). They uncover misconceptions about diabetes within the population that could impede preventive and curative programs as well as behavioral changes (27). Apart from serving as effective baselines for intervention programs, KAP studies inform the development of effective health education programs and techniques.

While KAP studies on diabetes have been conducted, additional research is essential, as inadequate KAP studies could heighten the risk of inadequate guidance and flawed intervention programs. This is especially needed for the Kingdom of Saudi Arabia (KSA), given its overburden healthcare system and a high prevalence of NCDs (29–31). The country ranks second in the Middle East and seventh worldwide in terms of diabetes prevalence (32). The lifestyle changes accompanying development have accentuated a higher incidence of obesity and increased susceptibility to diabetes in the country. Despite the severity of the situation, the majority of diabetes KAP studies have focused on either diabetes patients, specific regions or urban areas, or particular population subgroups. Findings from such studies may not consistently reflect the broader public accurately. This study seeks to contribute to the existing diabetes KAP literature by utilizing cross-sectional data collected from the general population of Saudi Arabia through self-reported questionnaires. By employing both univariate and multivariate regression data analyses, the study seeks to assess the level of diabetes KAP and how it varies across socio-demographic factors in Saudi Arabia. It is one of the few studies that offer a comprehensive examination, encompassing estimates from the general population, individuals with medical knowledge, and diabetes patients, while exploring variations across social and demographic factors.

## Methodology

### Study design and sample

This cross-sectional study was carried out targeting the general population of Saudi Arabia covering all administrative regions in the country, from 25 July 2023 to 5 September 2023. Data were collected online, via a self-reported questionnaire, using SurveyMonkey. Taking into account the widespread internet, and social media usage among individuals in Saudi Arabia (33), a link to the survey was shared with respondents through Twitter and WhatsApp groups. Participants were also requested to pass the link to their contact and ask them to participate in the study.

The study aimed to maximize reach and gather data from a wide range of respondents, as larger the target sample size, the higher the external validity and the greater the generalizability of the study (34). According to the most recent KSA census, the population of Saudi Arabia is 32,175,224 (35). To achieve the study’s objectives and ensure sufficient statistical power, a representative target sample size was calculated using a sample size calculator (36). The calculator determined that 385 participants would be needed, considering a margin of error of ±5%, a confidence level of 95%, a response distribution of 50%, and a population size of 32,175,224 individuals.

### Study instrument

The study used a validated questionnaire related to knowledge, attitudes, and practices regarding diabetes (37). The author B.A. translated the questionnaire from English to Arabic, then translated back to English by M.K.A to ensure the meaning of the content. The Arabic version of the questionnaire was used to collect the data.

Prior to proceeding with the online questionnaire, participants were presented with a clear explanation of the study’s purpose on the first page. They were explicitly notified that they had the freedom to withdraw from the study at any point without providing a reason. Additionally, participants were assured that all the information and opinions they provided would be treated as anonymous and kept confidential. To be eligible for participation, individuals had to be residents of Saudi Arabia, aged 18 years or older, understand the questionnaire’s content, and willingly agree to take part in the study. They were also required to have a proper understanding of the questionnaire’s content and. Online informed consent was obtained from participants before they were able to proceed with the questionnaire.

The questionnaire consisted of four primary sections. The first section gathered general information and information on respondents’ sociodemographic characteristics, including gender, age, marital status, education level, education related to medical, whether participants suffer from a diabetes, work status, and monthly income level. The second section assessed participants’ knowledge of diabetes. This section included items on risk factors, diagnosis, prevention, and complications of diabetes. The third section assessed participants’ attitudes toward diabetes using seven questions related to adherence to treatment of diabetes. The final section of the questionnaire assessed the respondents’ practices. This section consisted of four questions related practices related to diabetes.

### Dependent variables

To measure knowledge about diabetes, participants were presented with a series of knowledge items and asked to provide their responses. Incorrect or uncertain answers (marked as “do not know”) were assigned a score of zero, while correct responses were given a score of one. The total knowledge score ranged from zero to 29, with higher scores indicating a greater knowledge of diabetes. The internal reliability of the knowledge items was assessed using Cronbach’s α coefficient, which yielded a value of 0.84. This indicates a high level of internal reliability (38).

Attitudes were measured by calculating scores based on respondents’ answers to each attitudinal question. The total attitude scores ranged from zero to 7, with higher scores indicating more positive attitudes. The internal reliability of the attitudinal items was assessed using Cronbach’s α coefficient, which yielded a value of 0.76. This indicates a satisfactory level of internal reliability.

In the section on practices, respondents were asked to indicate “yes” or “no” in response to the items. A score of one was assigned to answers that reflected good practices, while a score of zero was assigned to answers that indicated otherwise. The total practice score ranged from zero to 4, with higher scores indicating positive practices.

### Independent variables

General information, socioeconomic and demographic characteristics including gender, age, marital status, education level, education related to medical, whether participants suffer from a diabetes, work status, and monthly income were used as independent variables. Gender was coded as a binary variable, where it takes the value of 1 if the respondent is men and 0 if women. The age variable was categorized into two groups: ≤40 years old (reference category), and ≥ 40 years old as an age of ≥40 is considered to be the typical age for the onset of diabetes (39). Marital status was recorded as a binary variable, with a value of one indicating marriage and zero indicating any other status. Education was divided into three categories: high school or below (reference category), college/university degree, and postgraduate degree. Education related to medical field where it takes the value of 1 if respondent has education related to medical field and 0 if not. Suffering from diabetes takes the takes the value of 1 if yes and 0 if not. Work status was broken down into unemployed (reference category), government employee, non-government employee, self-employed, students, and retiree. Monthly income, denominated in Saudi Riyal (SR) with an exchange rate of USD 1 = SR 3.75, was grouped into four categories: less than SR 7,000 (reference category), SR 7,000 to less than SR 10,000, SR 10,000 to less than SR 15,000, and SR 15,000 or more.

### Analysis methods

The data analysis in this study consisted primarily of univariate and multivariable regression analyses. Univariate analysis was employed to summarize the frequency distribution of social and demographic variables. To assess differences in mean values for knowledge, attitudes, and practice scores, one-way analysis of variance (ANOVA) was utilized.

Furthermore, a multivariable linear regression analysis was conducted to identify factors associated with knowledge, attitudes, and practice. This analysis aimed to determine the relationships between these variables and other relevant factors. STATA software (StataCorp LP, Texas, USA) was used to perform all the data analyses in this study.

### Ethical considerations

All procedures performed in this study, involving human participants, complied with the institutional and/or national research committee ethical standards, and the 1964 Helsinki declaration and subsequent amendments or equivalent ethical standards. This study has been reviewed and approved by the King Abdulaziz University Research Ethics Committee and was designed and performed in accordance with the ethical principles established by the university. Therefore, ethical approval was obtained from the Biomedical Ethics Research Committee, Faculty of Medicine, King Abdulaziz University (Ref-270-23). Online informed consent to participate was secured from all respondents who participated in the study. The data collection procedure was anonymous and as such no personal identifying information was collected.

## Results

### Social and demographic characteristics

A total of 646 participants completed the questionnaire. After excluding 29 respondents who did not provide complete answers for all the variables of interest, the final sample comprised 617 participants. The sociodemographic characteristics of the study participants are detailed in Table 1. The table shows that the mean diabetes knowledge score stood at 17.79 (SD = 5.39, ranging from 0 to 29), with an overall accuracy rate of 61.34% (calculated as 17.79 out of 29, multiplied by 100). Additionally, the mean attitude score regarding diabetes was 2.33 (SD = 1.91, ranging from 0 to 7), while the mean score for diabetes-related practices averaged at 2.58 (SD = 1.28, ranging from 0 to 4).

**Table 1:** Social and demographic characteristics of the study participants.

Variable	Mean	SD	Min	Max	*N*	%
Knowledge score	17.79	5.39	0	29		
Attitude score	2.33	1.91	0	7		
Practice score	2.58	1.28	0	4		
Gender						
Female					427	69.21
Male					190	30.79
Age						
< 40					326	52.84
≥ 40					291	47.16
Marital status						
Not married					244	39.55
Married					373	60.45
Education						
High school or below					149	24.15
College/University degree					350	56.73
Postgraduate degree					118	19.12
Education related to medical field						
No					431	69.85
Yes					186	30.15
Suffer from a diabetes						
No					554	89.79
Yes					63	10.21
Work status						
Unemployed					113	18.31
Government employee					284	46.03
Non-government employee					46	7.46
Self-employed					31	5.02
Student					78	12.64
Retiree					65	10.53
Monthly income						
< SR 7000					277	44.89
SR 7000 to <10,000					102	16.53
SR 10,000 to <15,000					127	20.58
≥ SR 15,000					111	17.99
Total					617	

Table 1 provides a comprehensive summary of the social and demographic characteristics of the study participants where the majority of the sample consisted of women (69.21%), married (60.45%), and did not have diabetes (89.79%). More than half of the participants (52.84%) were below 40 years of age, and the majority (56.73%) held a college or university degree. Respondents were categorized based on their monthly income, with 277 (44.89%) falling into the group earning less than SR 7,000, and 111 (17.99%) belonging to the group earning SR 15,000 or more. In terms of employment status, 113 (18.31%) were unemployed, and 65 (10.53%) were retired.

Tables 2–4 offer an overview of the responses related to KAP regarding diabetes. Table 2 provides a comprehensive breakdown of participants’ knowledge about specific diabetes-related topics and their understanding of how these issues operate and relate to the disease. In contrast, Table 3 outlines the attitudes held by participants toward diabetes, and Table 4 consolidates the practices in which participants engage concerning diabetes.

**Table 2:** Responses to the questionnaire on diabetes knowledge.

		*N* (%)	
		Correct answer	Incorrect answer
1.	What happens to blood sugar in diabetes?	415 (67.26)	202 (32.74)
2.	Dysfunction of which of the following organs leads primary to diabetes?		
2.1.	Lung	362 (58.67)	255 (41.33)
2.2.	Kidney	309 (50.08)	308 (49.92)
2.3.	Pancreas	542 (87.84)	75 (12.16)
2.4.	Liver	294 (47.65)	323 (52.35)
2.5.	Brain	313 (50.73)	304 (49.27)
3.	Is diabetes curable with treatment?	277 (36.79)	390 (63.21)
4.	Which of the followings are risk factors of diabetes?		
4.1.	Family history of diabetes mellitus	514 (83.31)	103 (16.69)
4.2.	Being overweight /Obesity	527 (85.41)	90 (14.59)
4.3.	Eating too much sugar	523 (84.76)	94 (15.24)
4.4.	Sedentary life (or not getting enough exercise)	378 (61.26)	239 (38.74)
4.5.	Stress	441 (71.47)	176 (28.53)
5.	Which of the followings are usual symptoms seen in diabetic patient?		
5.1.	Increased thirst	505 (81.85)	112 (18.15)
5.2.	Poor appetite	180 (29.17)	437 (70.83)
5.3.	Frequent urination	570 (92.38)	47 (7.62)
5.4.	Abdominal pain	221 (35.82)	396 (64.18)
5.5.	Palpitation (due to high blood sugar)	353 (57.21)	264 (42.79)
5.6.	Slow healing of cuts and wounds	539 (87.36)	78 (12.64)
6.	Which of the following therapies are effective in controlling blood sugar?		
6.1	Insulin injection	540 (87.52)	77 (12.48)
6.2.	Oral medications	346 (56.08)	271 (43.92)
6.3.	Regular exercise	518 (83.95)	99 (16.05)
6.4.	Avoiding sugary foods	516 (83.63)	101 (16.37)
6.5.	Regular eating of (herbs, ginger and cinnamon)	233 (37.76)	384 (62.24)
7.	Which of the following complications can occur due to diabetes?		
7.1.	Stroke	263 (42.63)	354 (57.37)
7.2.	Heart attack	278 (45.06)	339 (54.94)
7.3.	Hepatitis	121 (19.61)	496 (80.39)
7.4.	Kidney failure	347 (56.24)	270 (43.76)
7.5.	Arthritis	74 (11.99)	543 (88.01)
8.	What is the best way to diagnose diabetes?	530 (85.90)	87 (14.10)

**Table 3:** Response to attitudinal questions regarding diabetes.

		*N* (%)		
		Yes	No	Do not know
1.	Do you think that controlling glucose with diet alone is superior to that of controlling glucose with diet and medications?	284 (46.03)	190 (30.79)	143 (23.18)
2.	Can long term use of metformin cause kidney damage?	119 (19.29)	65 (10.53)	433 (70.18)
3.	Does long term drug use cause organ failure?	146 (23.66)	123 (19.94)	348 (56.40)
4.	Does insulin cause harmful effects to the body?	134 (21.72)	167 (27.07)	316 (51.22)
5.	Do you think the use of ginger, cinnamon, and fenugreek is better for treating diabetes than prescription drugs?	69 (11.18)	314 (50.89)	234 (37.93)
6.	Do you think that alternative therapies acupuncture, yoga, hypnosis, relaxation exercises or herbal remedies are better than the methods usually prescribed (diet control and medication)?	55 (8.91)	275 (44.57)	287 (46.52)
7.	Do you think there is no point in trying to get control of your blood sugar well, because the complications of diabetes will occur anyway?	79 (12.80)	301 (48.78)	237 (38.41)

**Table 4:** Practices related to diabetes.

	Statement	*N* (%)	
		Yes	No
1.	Would you consider treatment if you or one of your family members are found to have diabetes?	521 (84.44)	96 (15.56)
2.	Do you do 30–60 min physical activity daily? (e.g., brisk walking, house activities, climbing staircase).	372 (60.29)	245 (39.71)
3.	Do you check your blood sugar regularly (at least annually)?	297 (48.14)	320 (51.86)
4.	Do you try to avoid refine sugar/sugary foods?	400 (64.83)	217 (35.17)

Table 2 provides an overview of the responses pertaining to diabetes knowledge. It highlights the areas where much of the sample demonstrates a strong understanding of diabetes-related concepts and those areas where knowledge appears to be lacking. For instance, 67.26% of respondents exhibit a clear understanding of the dynamics of blood sugar in diabetes, while 32.74% lack this knowledge. In contrast, 63.21% of participants are uncertain about whether diabetes can be cured through treatment with only 36.79% of participants giving the correct responses. In terms of diabetes mellitus risk factors, a significant portion of the sample possesses knowledge about the association between risk factors such as a family history of diabetes (83.31%), being overweight or obese (85.41%), and excessive sugar consumption (84.76%). However, it’s worth noting that a considerable number of respondents are unaware that a sedentary lifestyle (38.74%) and stress (28.53%) can also contribute to the risk of diabetes.

Furthermore, Table 2 reveals some key insights. Notably, 87.52% acknowledge the effectiveness of insulin injections in controlling blood sugar levels, but a mere 37.76% are informed about the potential blood sugar-regulating properties of herbs, ginger, and cinnamon. Moreover, 54.94% of the participants are unaware that diabetes can lead to complications such as heart attack, and 43.76% lack knowledge about the connection between diabetes and kidney failure. However, the majority of participants (87.84%) understand the link between a dysfunctional pancreas and the development of diabetes. The table highlights specific areas where educational programs concerning diabetes could concentrate their efforts to enhance knowledge as a part of the broader process aimed at reducing the prevalence of diabetes mellitus.

Table 3 presents responses to questions that shed light on the attitudes of the participants toward diabetes. The majority of participants expressed uncertainty regarding whether long-term use of metformin might result in kidney damage (70.18%) and whether extended drug use could potentially lead to organ failure (56.40%). Additionally, 51.22% were unsure about whether insulin could have detrimental effects on the body. The percentage of “do not know” responses in Table 3 outweighs both the positive and negative responses in most the questions, suggesting that participants do not hold strong attitudes or opinions on these specific issues.

Table 4 provides insights into the practices related to diabetes among the participants. A substantial proportion of the sample (84.44%) express their willingness to consider treatment if they or a family member were diagnosed with diabetes, while only 15.56% indicate that they would not consider it. Additionally, a significant percentage (60.29%) engage in daily physical activities, such as brisk walking, house chores, or stair climbing for 30–60 min, although a notable portion (39.71%) do not partake in such activities. When it comes to regular blood sugar monitoring, only 48.14% of participants do so (at least annually), while the majority (51.86%) do not. On a positive note, a significant percentage (64.83%) try to avoid refined sugar and sugary foods, which is commendable.

### Differences in knowledge, attitudes, and practices toward diabetes

After examining the univariate statistics for each variable of interest, the study proceeded to further evaluate the disparities in the scores pertaining to KAP. The outcomes of this assessment are presented in Table 5.

**Table 5:** Comparison of social and demographic characteristics, and mean diabetes score.

Variable	*N*	%	Knowledge score			Attitude score			Practice score		
			Mean	SD	*p-*value	Mean	SD	*p*-value	Mean	SD	*p*-value
Gender					0.958			0.352			0.015**
Female	427	69.21	18.03	5.38		2.34	1.87		2.51	1.21	
Male	190	30.79	17.27	5.40		2.89	1.98		2.72	1.40	
Age					0.364			0.150			0.074*
<40	326	52.84	17.63	5.52		2.44	1.97		2.47	1.21	
≥40	291	47.16	17.97	5.24		2.19	1.81		2.69	1.34	
Marital status					0.029**			0.834			0.536
Not married	244	39.55	16.88	5.74		2.33	1.89		2.44	1.30	
Married	373	60.45	18.38	5.06		2.38	1.91		2.66	1.25	
Education					0.015**			0.104			0.009***
High school or below	149	24.15	15.93	6.04		2.02	1.77		2.40	1.46	
College/University degree	350	56.73	18.43	4.97		2.38	1.87		2.58	1.22	
Postgraduate degree	118	19.12	18.23	5.21		2.54	2.12		2.77	1.15	
Education related to medical field					0.004***			0.009***			0.246
No	431	69.85	17.16	5.57		2.01	1.75		2.50	1.30	
Yes	186	30.15	19.24	4.63		3.03	2.05		2.75	1.20	
Suffer from a diabetes					0.068*			0.920			0.139
No	554	89.79	17.67	5.46		2.29	1.90		2.51	1.28	
Yes	63	10.21	18.85	4.55		2.61	1.92		3.11	1.10	
Work status					0.053*			0.167			0.513
Unemployed	113	18.31	17.24	5.98		2.23	1.88		2.53	1.23	
Government employee	284	46.03	18.56	5.37		2.48	2.02		2.60	1.26	
Non-government employee	46	7.46	17.76	4.45		2.32	1.59		2.82	1.16	
Self-employed	31	5.02	17.86	4.44		2.37	1.97		2.51	1.59	
Student	78	12.64	16.01	5.63		2.23	1.84		2.21	1.26	
Retiree	65	10.53	17.47	4.52		1.89	1.62		2.83	1.29	
Monthly income					0.008***			0.283			0.088*
< SR 7000	277	44.89	16.89	5.80		2.23	1.92		2.42	1.35	
SR 7000 to <10,000	102	16.53	17.71	5.41		2.19	1.72		2.50	1.28	
SR 10,000 to <15,000	127	20.58	18.93	4.60		2.41	1.85		2.64	1.16	
≥ SR 15,000	111	17.99	18.79	4.74		2.55	2.07		2.94	1.13	

***p < 0.01, **p < 0.05, *p < 0.1.

Table 5 shows that, except for gender and age, knowledge scores exhibit statistically significant differences across all other variables. For example, significant disparities exist in the mean knowledge scores concerning marital status (*p* < 0.05), educational background (*p* < 0.05), education related to the medical field (*p* < 0.01), diabetes diagnosis (*p* < 0.1), work status (*p* < 0.1), and monthly income (*p* < 0.01). These disparities point to variations in diabetes-related knowledge among various demographic characteristics. However, the variations in knowledge scores do not correspond to differences in attitude toward diabetes, as indicated by the lack of statistical significance between attitude scores and most socio-demographic variables, except for those with an educational background related to the medical field (*p* < 0.01). A similar pattern emerges when considering diabetes-related practices, with significant differences being evident across gender (*p* < 0.05), age (*p* < 0.1), educational background (*p* < 0.01), and income (*p* < 0.1). These findings are further examined using regression analysis.

### Econometric results

In addition to the univariate and non-parametric analyses presented in earlier sections, the study also conducted regression analysis to further examine the KAP scores across several socio-demographic factors. Table 6 presents the results were higher scores indicate greater levels of knowledge, practices, and attitudes.

**Table 6:** Regression results of KAP-related factors for diabetes.

Variable	Knowledge	Attitude	Practice
	$\beta \;\left(SE\right)$	$\beta \;\left(SE\right)$	$\beta \;\left(SE\right)$
Gender			
Male	−1.135**	−0.073	0.069
	(0.506)	(0.183)	(0.123)
Age			
≥40	0.033	−0.060	0.102
	(0.582)	(0.206)	(0.142)
Marital status			
Married	1.244**	0.325*	0.102
	(0.520)	(0.189)	(0.131)
Education			
College/University degree	1.895***	0.190	0.171
	(0.629)	(0.190)	(0.148)
Postgraduate degree	1.436**	0.213	0.318*
	(0.778)	(0.254)	(0.179)
Education related to medical field			
Yes	1.997***	1.005***	0.303**
	(0.468)	(0.184)	(0.118)
Suffer from a diabetes			
Yes	1.564**	0.449*	0.567***
	(0.615)	(0.254)	(0.163)
Work status			
Government employee	−0.248	−0.113	−0.361
	(0.811)	(0.280)	(0.184)
Non-Government employee	−0.001	−0.091	0.120
	(0.905)	(0.316)	(0.223)
Self-employed	0.575	0.053	−0.258
	(0.930)	(0.440)	(0.304)
Student	−0.631	−0.047	−0.277
	(0.890)	(0.273)	(0.192)
Retiree	0.139	−0.310	0.040
	(0.992)	(0.333)	(0.240)
Monthly income			
SR 7000 to <10,000	0.545	0.014	0.078
	(0.668)	(0.256)	(0.171)
SR 10,000 to <15,000	1.161*	−0.010	0.212
	(0.659)	(0.266)	(0.172)
≥ SR 15,000	1.035*	0.080	0.441**
	(0.733)	(0.299)	(0.186)
*N*	617	617	617

***p < 0.01, **p < 0.05, *p < 0.1.

The results reveal variations in the levels of knowledge, attitudes, and practices among the population of the KSA, with certain socio-demographic variables showing significant associations, while others exhibit no significant impact. Table 6 demonstrates that there is no statistically significant difference in the levels of knowledge, attitudes, and practices related to diabetes when considering age and work status. This suggests that in matters concerning diabetes related KAP in Saudi Arabia, age, occupational status whether government, non-government, self-employed, student status, or retirement status have minimal bearing. However, Table 6 highlights significant differences in knowledge scores based on gender, marital status, educational background, medical field-related education, diabetes diagnosis, and income.

The findings indicate that males exhibit lower levels of knowledge (*β* = −1.135; *p* < 0.05) compared to females, while married individuals demonstrate higher levels of knowledge (*β* = 1.244; *p* < 0.05) in comparison to those who are unmarried. Likewise, Table 6 illustrates that possessing a college or university degree is linked to higher knowledge scores (*β* = 1.895; *p* < 0.01), in a similar manner, to having a postgraduate degree (*β* = 1.436; *p* < 0.05). Similarly, the pattern holds true for individuals with an education linked to a medical field and those experiencing diabetes. Those with a medical field-related education exhibit significantly higher levels of knowledge (*β* = 1.997; *p* < 0.01) compared to those without such an educational background. Likewise, individuals suffering from diabetes demonstrate more knowledge (*β* = 1.564; *p* < 0.05) compared to those without the condition. Furthermore, knowledge of diabetes is correlated with income, showing a significant disparity between participants with higher monthly income levels (*β* = 1.161; *p* < 0.1; *β* = 1.035; *p* < 0.1) and those with lower income levels, indicating that individuals with higher income possess greater knowledge.

When it comes to attitudes toward diabetes, significant differences are not observed across most socio-demographic variables, except in the cases of marital status, educational background related to the medical field, and those individuals suffering from diabetes. Table 6 shows that being married is linked to a more positive attitude (*β* = 0.325; *p* < 0.1) when compared to being unmarried. Similarly, having an educational background related to the medical field significantly enhances one’s attitude toward diabetes (*β* = 1.005; *p* < 0.01) in contrast to those without such an education. Furthermore, experiencing diabetes is associated with a more favorable attitude (*β* = 0.449; *p* < 0.1) compared to individuals who are not suffering from the disease.

Regarding diabetes-related practices, having a postgraduate degree (*β* = 0.318; *p* < 0.1), an educational background related to the medical field (*β* = 0.303; *p* < 0.05), living with diabetes (*β* = 0.567; *p* < 0.01), and having higher income levels (*β* = 0.441; *p* < 0.05) are all positively associated with the adoption of positive practices. In summary, as shown in Table 6, individuals with a medical background and those grappling with diabetes exhibit higher levels of KAP related to diabetes among the Saudi Arabian population. Moreover, there exists a direct correlation between higher levels of education and income and enhanced knowledge and practices related to diabetes.

## Discussion

Recognizing the significance of self-care in diabetes management, this study employed cross-sectional data from the general population of the KSA to examine KAP of diabetes and their variations across socio-demographic factors in the Kingdom. Both univariate and multivariate analyses show that individuals suffering from diabetes exhibit higher KAP levels while those with a medical education demonstrate significantly greater knowledge (*β* = 1.997; *p* < 0.01), more positive attitudes (*β* = 1.005; *p* < 0.01), and better practices (*β* = 0.303; *p* < 0.05) related to diabetes. Furthermore, an increase in education and income is associated with higher knowledge and better diabetes management practices. In contrast to some prior research (40, 41), age and work status did not show significant differences in KAP related to diabetes. To enhance knowledge, attitudes, and practices toward diabetes management, the government should implement educational programs tailored to the relevant socio-demographic differences.

Drawing direct comparisons with existing literature can be challenging due to the varying focus on specific population segments and regions. However, general comparisons can still be made, particularly concerning socio-demographic characteristics. For example, both univariate and multivariate analyses indicated that married individuals, as compared to unmarried ones, exhibit higher knowledge (*β* = 1.244; *p* < 0.05) and more positive attitudes (*β* = 0.325; *p* < 0.1). Similar findings that linked marriage to increased knowledge and more positive attitudes have been reported in other studies (42, 43). This could be attributed to the fact that married individuals are more likely to have at least one family member who might be affected by the disease, unlike unmarried individuals. Existing literature suggests that those with a family history of diabetes tend to possess greater knowledge and exhibit more positive attitudes toward the condition (44, 45).

The observation that higher education correlates with increased knowledge is a recurring theme in literature. Consistent findings have been reported in various studies, such as those in Bangladesh (46), the Hail region of Saudi Arabia (47), and among diabetes patients in Nepal (48). Furthermore, this study has found that those with education related to medical field have higher knowledge (*β* = 1.997; *p* < 0.01), higher positive attitudes (*β* = 1.005; *p* < 0.01) and good practices (*β* = 0.303; *p* < 0.05) related to diabetes. The higher KAP score is likely a result of higher exposure to comprehensive diabetes information which, in turn, contributes to a more positive attitude (49). Self-care education is crucial for effective diabetes management as it helps correct misconceptions regarding diabetes treatment and management (37). Therefore, while there are multiple avenues to improve KAP regarding diabetes, one effective approach is providing training, workshops, and educational programs for those with low levels of education in Saudi Arabia.

The higher KAP scores related to diabetes among individuals with medical education also apply to diabetes patients. The findings reveal that diabetes patients possess more knowledge and exhibit a more positive attitude toward diabetes compared to those without the condition. Similar findings in other studies indicate that individuals who have either been diagnosed with the disease or have a family member with the condition tend to demonstrate greater knowledge, maintain a positive attitude, and engage in better practices, as they are exposed to more information while seeking to aid themselves or their relatives (6, 40).

In addition to higher education resulting in higher knowledge scores, the study also found that increased income was associated with greater level of knowledge about diabetes. This is possibly because individuals with higher income levels are likely to engage in sedentary lifestyles which could contribute to high diabetes prevalence, thereby increasing their likelihood of having more knowledge about the disease (41). Additionally, Gillani et al. (50) underscores that individuals with ample financial resources tend to exhibit more positive attitudes toward seeking treatment. In contrast, individuals with limited means require support and encouragement to enhance their participation. Financial constraints are linked to negative attitudes, highlighting the potential significance of family income in efforts to shape attitude changes (46).

The relationship between gender and KAP concerning diabetes has yielded mixed findings in the literature. While some studies have reported higher knowledge scores among males, as observed in Al Hasa district, Saudi Arabia (51), the United Arab Emirates (52), and Pakistan (50), it’s important to note that these results are not consistent across all studies. For example, this study, in contrast, has identified that males exhibit lower knowledge about diabetes compared to females (*β* = −1.135; *p* < 0.05). This could be attributed to the fact that women with higher knowledge about diabetes are selected in the sample. This observation aligns with similar findings in Qatar (8) and among university students in Jeddah, Saudi Arabia (53), where females demonstrated a stronger understanding of diabetes. Nevertheless, concerning diabetes management practices, the study revealed that males exhibited better practices than females, aligning with findings from Aljofan et al. (47) in Saudi Arabia and Gautam et al. (48) in Nepal. This could be attributed to the greater willingness of male participants to make lifestyle adjustments compared to females.

The study has certain limitations worth noting. Firstly, it relies on self-reported data, which can be susceptible to biases. For instance, the survey does not delve into how participants accessed diabetes information, which could be valuable for devising targeted publicity strategies for the disease. Additionally, while the study includes the general population, the sample consists of only 617 participants, potentially limiting the generalizability of the findings to the entire Saudi Arabian population. This sample size was constrained by time and resource limitations. Furthermore, the study’s design and sampling technique do not establish causality, so the results should be interpreted as associations rather than causal relationships. It’s also important to acknowledge that data collection occurred online, potentially excluding individuals who are not familiar with online surveys, and responses may be skewed toward those with higher levels of education and income. However, despite these constraints, the study offers valuable insights into knowledge, attitudes, and practices related to diabetes, which can inform the development of diabetes intervention and prevention strategies.

## Conclusion

This study used cross-sectional data to examine knowledge, attitudes, and practices of diabetes among the population of Saudi Arabia and assess their variations across socio-demographic factors. Through univariate and multivariate analyses, the study reveals distinct variations in knowledge, attitudes, and practices of diabetes among participants based on their gender, education, marital status, income, diabetes patient status, and having a medical field-related education. Although knowledge scores are relatively high, attitudes and practices related to diabetes generally lag behind, highlighting the need to implement strategies that promote changes in attitude and healthy lifestyle practices to mitigate diabetes-related risks. The findings also suggest a necessity for heightened care and specialized educational programs for individuals who are unmarried, lack a medical field-related education, and have lower levels of education and income. As diabetes management heavily relies on self-care, the government should prioritize improving diabetes KAP scores to prevent additional complications and burdens that could strain the healthcare system.

## Data availability statement

The datasets generated and/or analysed during the current study are not publicly available due to privacy and confidentiality agreements as well as other restrictions but are available from the corresponding author on reasonable request.

## Ethics statement

All procedures performed in this study, involving human participants, complied with the institutional and/or national research committee ethical standards, and the 1964 Helsinki declaration and subsequent amendments or equivalent ethical standards. This study has been reviewed and approved by the King Abdulaziz University Research Ethics Committee and was designed and performed in accordance with the ethical principles established by the university. Therefore, ethical approval was obtained from the Biomedical Ethics Research Committee, Faculty of Medicine, King Abdulaziz University (Ref-270-23). Online informed consent to participate was secured from all respondents who participated in the study. The data collection procedure was anonymous and as such no personal identifying information was collected.

## Author contributions

BA-W: Conceptualization, Data curation, Investigation, Methodology, Validation, Writing – original draft, Writing – review & editing. MA-H: Conceptualization, Data curation, Formal analysis, Investigation, Methodology, Software, Validation, Writing – original draft, Writing – review & editing, Project administration, Supervision.
